# Manufacture of Platform Chemicals from Pine Wood Polysaccharides in Media Containing Acidic Ionic Liquids

**DOI:** 10.3390/polym12061215

**Published:** 2020-05-27

**Authors:** Mar López, Carlos Vila, Valentín Santos, Juan Carlos Parajó

**Affiliations:** Chemical Engineering Department, University of Vigo (Campus Ourense), Polytechnical Building, As Lagoas, 32004 Ourense, Spain; marlopezr@uvigo.es (M.L.); cvila@uvigo.es (C.V.); vsantos@uvigo.es (V.S.)

**Keywords:** cellulose, hemicelluloses, wood polysaccharides, platform chemicals, acidic ionic liquid

## Abstract

*Pinus pinaster* wood samples were subjected to chemical processing for manufacturing furans and organic acids from the polysaccharide fractions (cellulose and hemicellulose). The operation was performed in a single reaction stage at 180 or 190 °C, using a microwave reactor. The reaction media contained wood, water, methyl isobutyl ketone, and an acidic ionic liquid, which acted as a catalyst. In media catalyzed with 1-butyl-3-methylimidazolium hydrogen sulfate, up to 60.5% pentosan conversion into furfural was achieved, but the conversions of cellulose and (galacto) glucomannan in levulinic acid were low. Improved results were achieved when AILs bearing a sulfonated alkyl chain were employed as catalysts. In media containing 1-(3-sulfopropyl)-3-methylimidazolium hydrogen sulfate as a catalyst, near quantitative conversion of pentosans into furfural was achieved at a short reaction time (7.5 min), together with 32.8% conversion of hexosans into levulinic acid. Longer reaction times improved the production of organic acids, but resulted in some furfural consumption. A similar reaction pattern was observed in experiments using 1-(3-sulfobutyl)-3-methylimidazolium hydrogen sulfate as a catalyst.

## 1. Introduction

How to supply humanity with enough food, energy, chemicals, and materials sustainably, without damaging our planet, is an unavoidable issue [1]. Lignocellulosic biomass (LB), mainly made up of lignin and polysaccharides (hemicelluloses and cellulose), is the most abundant source of organic carbon, and represents a key resource for the integrated production of chemicals, materials, and energy [2]. Based on its renewable nature, widespread occurrence, and huge availability, LB holds significant potential as a raw material for the sustainable production of chemicals, fuels, and materials. Owing to its carbon-neutral character, LB has been considered a major part of carbon mitigation strategies [3].

Compared with other types of LB, wood shows diverse advantages as feedstock for the industry, including low production cost, easy storage, non-seasonal character, high cellulose content and large availability. Pine trees are evergreen conifers widely distributed in extensive geographic areas. *Pinus pinaster*, a major forest resource in France and the Iberian Peninsula, has been considered as a raw material for the production of bio-based chemicals in the scope of biorefineries [4,5,6,7,8,9,10,11,12]. As with other types of LB, the composition of *Pinus pinaster* wood is usually reported in terms of non-structural and structural components. Typically, the non-structural components (extractives, ash, proteins, etc.) account for about 10 wt% of pine wood, whereas the structural components include the following polymers:

○ Lignin, a phenolic fraction that (as in other softwood species) is difficult to deconstruct, owing to the predominance of guayacil structural units [13].
○ Cellulose, a linear homopolymer made up of d-anhydroglucose units bound through β-1-4 glycosidic linkages.
○ Hemicelluloses, which include two major types of polymers: glucomannan (with a backbone made up of glucose and mannose structural units, with acetyl and galactosyl substituents) and arabinoxylans [14,15].

The structural components appear in the LB cell wall as interpenetrated polymers, configuring a complex and heterogeneous structure responsible for the recalcitrance of the feedstocks to chemical and enzymatic processing. Achieving high product yields from the polymeric constituents of wood in an integrated biorefinery process is an approach with great potential [16], but the recalcitrance of the feedstock makes it a tremendous challenge [2]. In fact, wood polymers are insoluble in most common industrial solvents, due to the strong intermolecular and intramolecular networks of hydrogen bonds and complex microstructure [17].

Separation and/or conversion of *Pinus pinaster* wood polymers can be achieved by aqueous processing in the presence of an acidic catalyst. In this type of treatment, lignin remains in solid phase, whereas the polysaccharides may undergo (depending on the operational conditions) one or more of the following reactions [4,6]:

○ acetyl groups hydrolysis into acetic acid,
○ hemicellulose hydrolysis into low molecular weight polymers, oligomers or sugars,
○ cellulose hydrolysis into oligomeric compounds or glucose,
○ dehydration or hydrolysis-dehydration of the soluble saccharides coming from polysaccharides into furans: furfural (F) and 5-hydroxymethyl furfural (HMF) can be obtained from pentoses and hexoses, respectively,
○ rehydration of HMF into levulinic acid and formic acid.

**Figure 1:**
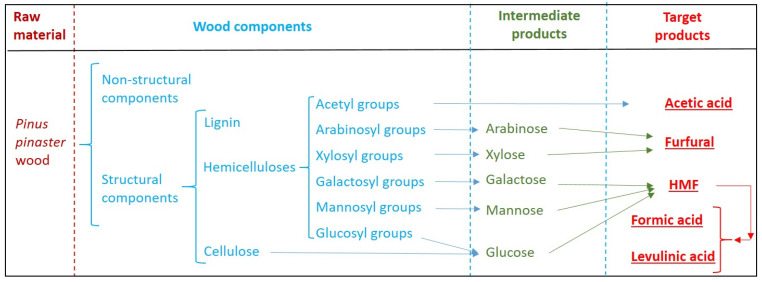
Scheme followed in this work for producing platform chemicals from *Pinus pinaster* wood polysaccharides.

This approach (summarized in Figure 1) provides a simple method for converting both cellulose and hemicelluloses into furans and organic acids.

A number of the reaction products in Figure 1 are important platform chemicals with a bright future, owing to emergent applications in the fields of polymers and renewable fuels. For example, it can be noted that:

○ Furfural can be employed to produce both alkanes (by aldol condensation with acetone followed by hydrogenation) and furanic biofuels such as methyltetrahydrofuran and tetrahydrofuran [18,19].
○ HMF may undergo deoxygenation and further reactions leading to furanic biofuels (such as 2,5-dimethylfuran and 2,5-dimethyltetrahydrofuran) [20,21], or to mixed alkanes for P-series fuels [19].
○ Levulinic acid, obtained by rehydration of HMF, can be employed in the manufacture of succinic acid, resins, polymers, herbicides, and pharmaceuticals [22], or hydrogenated into γ-valerolactone (GVL), a solvent suitable as a gasoline extender [23].
○ Formic acid is a commercial solvent that can also be suitable as a hydrogen donor for the reduction of levulinic acid into GVL [24,25], or as a fuel in direct formic acid fuel cells (DFAFC) [26,27].

However, this simplified sequence of reactions represents just a part of the complex reaction mechanism describing the formation of target products from pine wood polysaccharides, which also includes a number of series and parallel parasitic reactions. For example, the formation of non-hydrolyzable saccharides from cellulose [28] is one of the parallel, non-productive reactions. On the other hand, the dehydration of hexoses and pentoses into furans proceeds through a series of reactive intermediates that may participate in the formation of undesired humin-type products [22,29,30,31,32]. Humins are a blackish charred material resulting from the cross-condensation/polymerization of aldehydic moiety of reactive species [33]. Although humins are considered waste products, their utilization as a feedstock for pyrolysis or steam reforming have been considered in literature [34,35]. Additional insight into the formation of humins can be found in the studies from Patil et al. [36]. Other influential reactions include the generation of furfural from hexoses by fragmentation reactions [37], and/or its conversion into formic acid [22].

Besides the participation of target products and intermediates in undesired reactions, the different susceptibility to hydrolysis-dehydration of cellulose and hemicellulosic polymers [10] is a key factor hindering the simultaneous production of target products derived from them. For example, the hemicellulosic polymers (particularly xylan) can be completely hydrolyzed when the cellulose conversion into glucose is far from being complete, and for this reason, the experimental yields of the target products can be significantly lower than the stoichiometric ones [8].

A number of strategies have been proposed to limit the effect of unwanted side reactions. For example, in biphasic aqueous/solvent media, some target products can be transferred to the organic phase, preventing their participation in unwanted reactions taking place in the aqueous phase; whereas this operation in energy-efficient reactors enabling fast heating profiles (such as microwave-heated reactors) results in improved reaction efficiency. Specifically, the combined utilization of water as a solvent and microwave irradiation has been considered as an efficient method enabling significant savings of energy and time [38,39]. Additionally, the results can be improved by using green, efficient, and selective catalysts, including ionic liquids [40,41,42,43,44].

The one-pot strategy for lignocellulose conversion shows a number of advantages over multistage processing, such as higher simplicity, minimization of down-stream operations and solvent utilization, easier isolation/purification of the target products, and decreased reaction times and labor-related resources [33].

In single-phase reactions, decreased furfural yields are obtained under conditions leading to the maximum concentrations of HMF and/or levulinic acid, because of their different kinetics of generation and consumption. In this study, in order to improve the simultaneous recovery of furfural and HMF/levulinic acid, the *Pinus pinaster* wood samples have been treated in the presence of methyl isobutyl ketone (MIBK), which shows favorable features as an extracting agent, including a “green” character [45], and a limited solubility in water. Additionally, MIBK shows a comparatively high partition coefficient for furfural, enabling its simultaneous separation/purification from the reaction media along the reaction. In order to improve the yields of the target products, the operation was carried out in a stirred, microwave-heated reactor.

Ionic liquids (ILs) are “green” salts with low melting temperatures (below 100 °C) and unique properties, such as very low vapor pressure, high thermal and chemical stability, non-flammability, chemical tunability, and a broad electrochemical window [17]. ILs might play a number of roles in biorefineries, for example as separation agents, reaction media and/or catalysts [46,47,48]. In particular, acidic ionic liquids (here denoted AIL) may perform as catalysts with improved activity, selectivity, and stability; and allowing easier separation and reuse [1]. AILs have been proposed as catalysts to achieve the conversion of pine wood polysaccharides. The catalysts employed in this study (1-butyl-3-methylimidazolium hydrogen sulfate, denoted [C4mim]HSO_4_, 1-(3-sulfopropyl)-3-methylimidazolium hydrogen sulfate, denoted [C3SO_3_Hmim]HSO_4_, and 1-(3-sulfobutyl)-3-methylimidazolium hydrogen sulfate, denoted [C4SO_3_Hmim]HSO_4_]) are made up of the hydrogensulfate anion and an alkyl- or sulfoalkyl-imidazolium cation. All the AILs employed in this study have been employed for LB processing with diverse objectives [10,42,44,47,49,50,51]. It can be noted that pine wood processing in biphasic media using AILs as catalysts results in the simultaneous generation and separation of furfural (which appears in the organic phase), in the generation of organic acids of low acidity (which are distributed between the organic and aqueous phases), and in partial lignin breakdown (yielding MIBK-soluble lignin fragments). The rest of the lignin remains in solid phase, together with residual polysaccharides (which appear in low percentages under conditions of practical interest).

Scarce information has been reported on the one-stage production of platform chemicals from the hemicellulosic and cellulosic fractions in native pine woods. Zhang and Zhao [52] obtained furfural and HMF (at 4.4–31% and 2.1–52% molar conversions, respectively) from pine wood in media containing [C4mim]Cl or [C_4_mim]Br in the presence of a Lewis acid (CrCl_3_) as a catalyst. In a related study, Sievers et al. [53] employed the same IL catalyzed with trifluoroacetic acid to obtain water-soluble products (monosaccharides, oligosaccharides, furfural, and HMF) from pine wood.

AILs have a large potential in replacing conventional acidic catalysts [50]. For a given cation, the anionic counter part of the IL greatly affects the catalytic activity, playing a vital role in the destruction of inter- and intramolecular H-bonds and hydrolysis of cellulose [33]. Among AILs, the ones containing the hydrogen sulfate anion exhibit a universal activity for the conversion of fructose, glucose, and cellulose into levulinic acid [44].

This work deals with the manufacture of furans and organic acids (furfural, HMF, acetic acid, levulinic acid and formic acid) from the hemicellulosic and cellulosic fractions of native *Pinus pinaster* wood, operating in single-stage mode in media containing water, MIBK and an AIL acting as a catalyst. The operation was carried out at 180 or 190 °C in a microwave-heated reactor, and the experimental results are discussed in terms of molar conversions and yields of the target products.

## 2. Materials and Methods

### 2.1. Raw Material and Reaction

*Pinus pinaster* wood chips were obtained from a local industry, milled to a particle size below 8 mm, air-dried, homogenized, and stored until use. Wood samples (2.00 g, oven-dry basis) were mixed with the desired amounts of distilled water (11.8 g), MIBK (24.0 g) and one of the considered AIL (0.200 g of [C4mim]HSO_4_, [C3SO_3_Hmim]HSO_4_ or [C4SO_3_Hmim]HSO_4_), and the mixtures were reacted in a stirred MARS 6 MW reactor under different reaction conditions (180 or 190 °C, reaction times in the range 7.5–30 min).

### 2.2. Analysis

Wood samples were analyzed for moisture, extractives, ash, and Klason lignin using NREL standard methods. Cellulose, acetyl groups and hemicellulose-derived sugars were determined by quantitative acid hydrolysis (NREL/TP-510-42618 method) followed by HPLC determination of the resulting sugars [9]. The insoluble solid residue obtained after the quantitative acid hydrolysis was considered as Klason lignin. Acetyl groups were calculated from the amount of acetic acid released upon quantitative acid hydrolysis. Samples of the organic and aqueous phases were analyzed by the HPLC method described elsewhere [9]. Reactions were carried out in biphasic media and performed in duplicate, and the mean values were reported. After reaction, the solid and liquid phases were separated by vacuum filtration. The treated solids were employed for solid yield determination. The organic and aqueous phases were allowed to separate by decantation and subjected to HPLC analysis, using the same method mentioned above.

### 2.3. Definition of Variables

The results are discussed in terms of molar conversions and product yield, which were calculated as follows:

Molar conversion (for product j) = 100 (mol product j in the reaction medium or in the considered phase)/mol of the correspondent precursor in the wood sample fed to the reactor, where arabinosyl and xylosyl groups were the furfural precursors (1 mol of furfural per mol of precursor); glucosyl, galactosyl and mannosyl groups were the precursors of HMF, levulinic acid and formic acid (1 mol anhydrohexose per mol or HMF, levulinic acid or formic acid); and acetyl groups were the precursors of acetic acid.

Product yield (for product j): 100·(g product I in the reaction medium or in the considered phase)/potential amount of product j in the wood sample fed to the reactor, where the potential amount of product j corresponded to the one resulting from the quantitative conversion of the precursor(s) into product j.

### 2.4. Error Assessment

The temperature uncertainty was 1 °C. The errors in the HPLC analyses were reported in terms of the absolute values of the deviations with respect to the respective means, expressed as percentages, and denoted ε. The value of ε depended on the concentration range, reaching values of 0.84, 0.92, and 1.87% for glucose, mannose and arabinose, respectively. The values of ε determined for the rest of the target compounds were as follows: 1.07, 1.3, 0.7, 0.6, 0.73, 0.6, and 1.03% for xylose, galactose, formic acid, acetic acid, levulinic acid, HMF, and furfural, respectively.

## 3. Results and Discussion

### 3.1. Composition of Pinus Pinaster

The choice of a given type of LB for industrial utilization is of paramount importance from both techno- and socio-economical points of view [48]. For practical purposes, the most important compositional data are the content of the structural components (lignin, cellulose, and hemicelluloses), since limited added-value can be reached from the non-structural components in most cases. Additionally, the relative abundance of syringyl and guayacil structural units in lignin, and the type and distribution of anhydrosugar units in hemicelluloses can be important for specific applications. For example, in the case of pine wood, the lignin (rich in guayacil units) is suitable for vanillin manufacture; whereas the hemicelluloses contain both C6 polymers (mannans, typically classified as glucomannan or galactoglucomannan, depending on the content of galactosyl groups) and C5 polymers (arabinoxylan) [15]. The compositional data determined for the wood samples employed in the experiments are listed in Table 1. The results determined for the diverse polysaccharides were expressed in terms of the respective anhydrosugar constituents, since the glucosyl units were present in both cellulose and hemicelluloses. For the purposes of this study, the most relevant data were the contents of acetyl groups (precursors of acetic acid), anhydropentoses (arabinosyl and xylosyl units, which were potential substrates for furfural production) and anhydrohexoses (glucosyl, mannosyl and galactosyl groups, which were potential substrates for HMF and/or levulinic acid/formic acid). The experimental results determined in this study were typical for pine wood, and lie in the ranges reported for *Pinus pinaster* [4,5,7,8,10,11,12], *Pinus taeda* [53], *Pinus sylvestris* [13,47] and *Pinus* spp. from China [54].

### 3.2. Experiments Using [C4mim]HSO_4_ as a Catalyst

Alkyl-imidazolium ionic liquids have been employed for processing pine wood (or fractions derived from its processing) in the field of biorefineries, acting as reaction media and/or catalysts. In general, aprotic imidazolium ionic liquids have been employed to process pine wood, eventually in the presence or absence of catalysts and co-solvents, to achieve a number of effects, including cellulose dissolution/regeneration and/or production of substrates susceptible to enzymatic hydrolysis, or hydrolysis-dehydration of polysaccharides [10,13,46,55,56,57,58,59,60,61,62,63,64,65,66,67,68,69].

Additionally, acidic alkyl-imidazolium ionic liquids have been employed for pine wood processing aiming at delignification, fractionation, and/or the manufacture of solids susceptible to enzymatic hydrolysis [10,47,70,71] in the absence of additional catalysts. Good results have also been obtained in applications such as reaction media or catalysts for the hydrolysis/dehydration of hemicellulose-derived saccharides, or for achieving the solubilization and/or conversion of lignin and polysaccharides present in native, dewaxed or hemicellulose-free pine wood [10,72]. In particular, alkyl imidazolium ILs bearing the HSO_4_^-^ anion (such as [C4mim]HSO_4_) have been successfully employed for lignin removal/fractionation of hemicellulose-free pine wood [10] or native pine wood [55,71], or for HMF manufacture from hemicellulose-derived saccharides resulting from hydrothermal processing [6,9,10]. Based on this information, [C4mim]HSO_4_ was selected as a catalyst for the first set of experiments.

The utilization of biphasic media for manufacturing furans and/or organic acids from LB has been proposed in literature for improving the results obtained in single-phase treatments. Although most information dealing with the utilization of biphasic media for producing furans and/or organic acids has been reported for aqueous media supplemented with mineral acids, some studies dealing with ILs are available. Among the available solvents, MIBK is a preferred candidate due to its stability toward acids, medium polarity, lower toxicity, average boiling point, poor miscibility with water, good extraction ability for polar products [49], and green character [45]. For example, MIBK has been employed in the formulation of reaction media, or to separate the target products after reaction [49,50,73]. Based on this information, MIBK was selected as a solvent for this set of experiments.

The target product yields are strongly dependent on a number of operational parameters, including the solid charge and temperature. The solid charges employed in literature studies vary within wide ranges. It is well-known that increased dilution results in improved yields, owing to the lower concentrations of intermediates involved in side reactions. In this study, the relative amount of solid was fixed in 1 g oven-dry wood/6 g of aqueous phase, which is among the highest reported in the field. The choice of the reactor (heated by microwaves) and the temperature range (180–190 °C) was based on the assumption that operation at higher reaction temperatures for short reaction times is favorable for levulinic acid formation [50].

**Figure 2:**
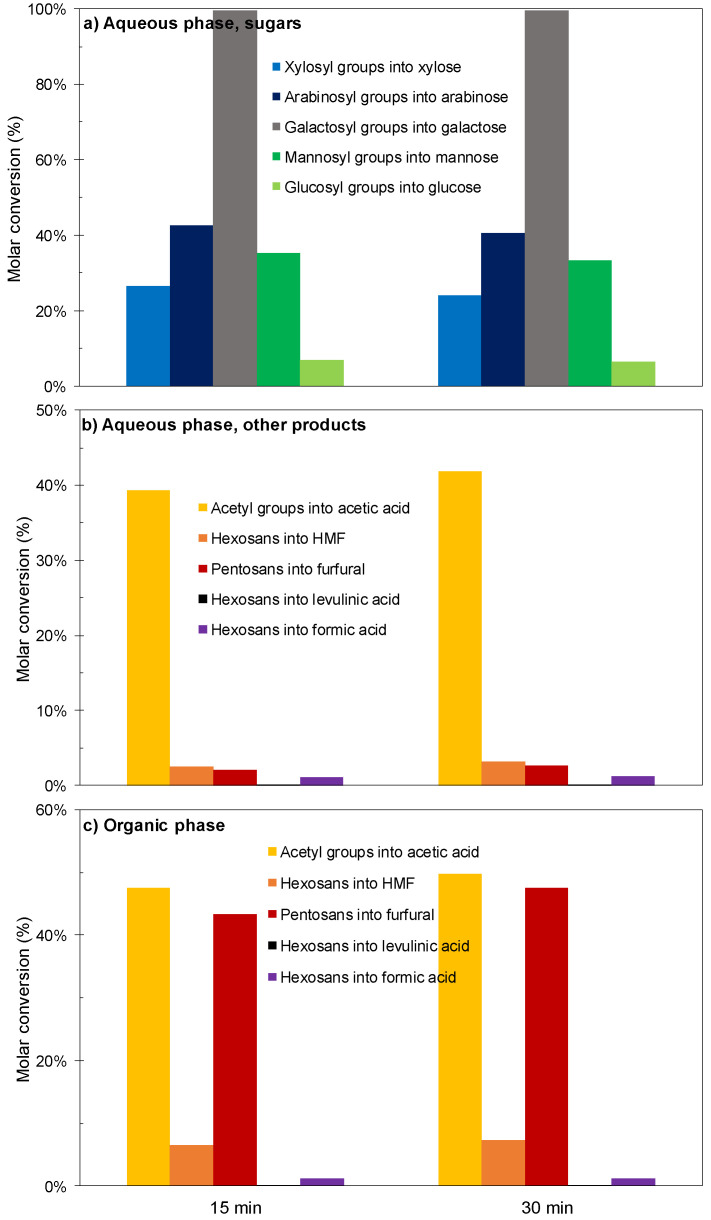
Results achieved in experiments performed at 180 °C using [C4mim]HSO_4_ as a catalyst. (**a**) Sugars in aqueous phase. (**b**) Other compounds in aqueous phase. (**c**) Compounds in organic phase.

**Figure 3:**
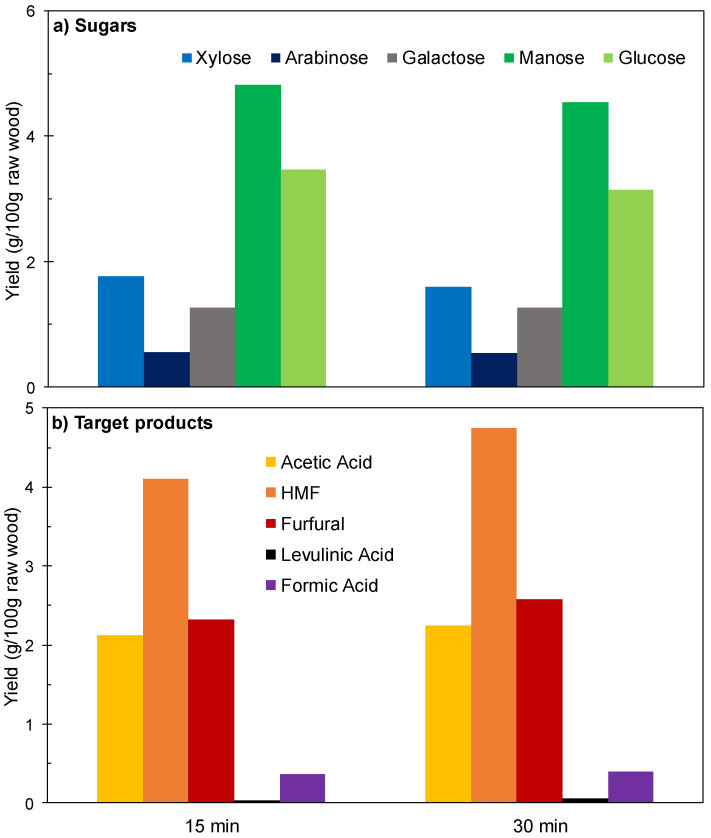
Overall yields (including the contributions of aqueous and organic phases) achieved in experiments performed at 180 °C using [C4mim]HSO_4_ as a catalyst. (**a**) Sugars. (**b**) Target products.

Figure 2 and Figure 3 show the experimental results achieved in the experiments performed under the conditions indicated above at reaction times of 15 or 30 min. The sugars coming from wood polysaccharides were concentrated in the aqueous phase (see Figure 2a), where galactose was obtained at near total conversion, revealing both a high susceptibility of galactosyl groups to hydrolysis and a limited susceptibility of galactose to consumption reactions.

In comparison, lower conversions were obtained for mannose (coming from the backbone of glucomannan) and for glucose (for which the precursors are both glucomannan and cellulose). Considering the scarce HMF production (Figure 2b), it can be concluded that the severity of the experimental conditions was not enough to cause the hydrolysis-dehydration of the glucomannan backbone. The solid yield recovery determined for these assays (57.1 and 57.7 g/100 g wood) was in agreement with this hypothesis. The molar conversions of pentosan into xylose and arabinose (in the range 23.9–42.4%) are comparatively low because a part of these sugars has been converted into furfural under the considered conditions. Oppositely, the target products (acetic acid, furfural, HMF, formic acid and levulinic acid) were distributed between phases (see Figure 2b,c). High molar conversion of acetyl groups into acetic acid was achieved at the two reaction times considered (the overall conversion calculated from the amounts contained in aqueous and organic phases exceeded 85%). Acetic acid was predominantly distributed in the aqueous phase (distribution coefficient, 0.480). Furfural was obtained at 45.3–50.1% molar conversion (including the amounts present in both phases), and appeared concentrated in the organic phase (distribution coefficient, 7.99), confirming the excellent ability of MIBK as a solvent for furfural extraction. However, low, or very low conversions were obtained for HMF and its rehydration products (formic and levulinic acids). The overall yields of the various products (including the amounts present in the organic and aqueous phases) are shown in Figure 3. Taking into account the limited conversions and yields obtained for furans and organic acids, as well as the distribution of the diverse reaction products, it can be inferred that higher reaction severities could result in improved results.

**Figure 4:**
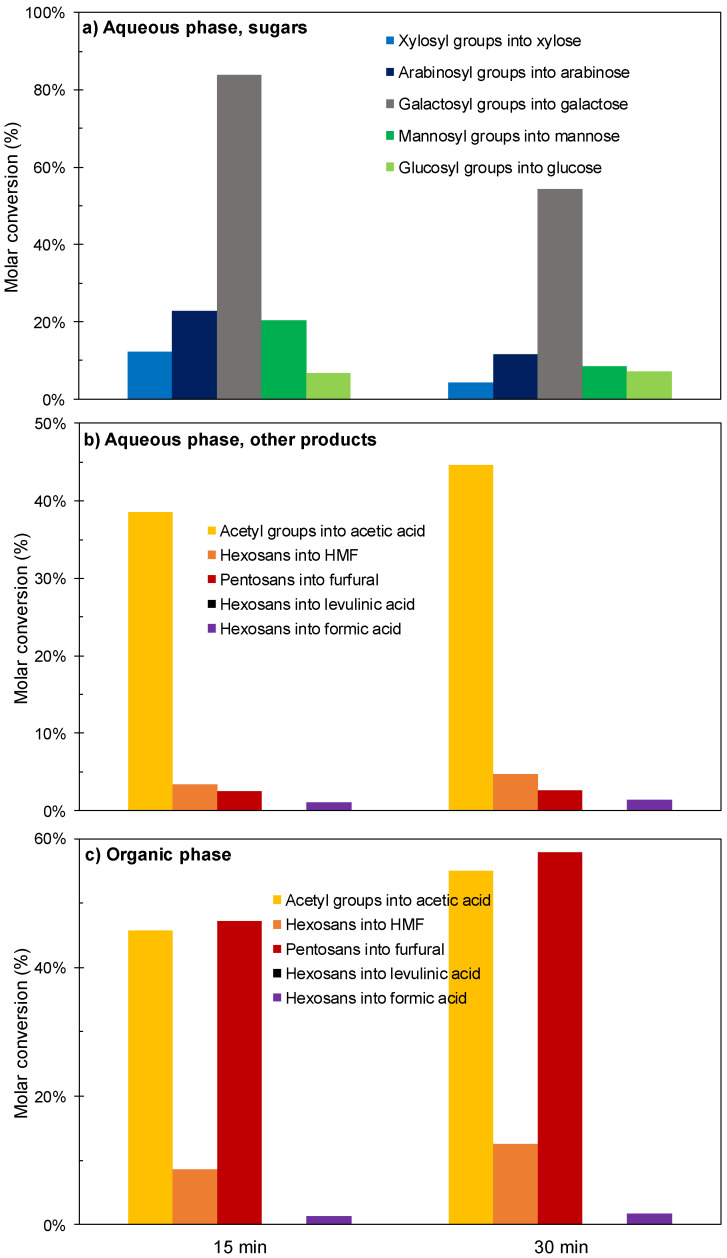
Results achieved in experiments performed at 190 °C using [C4mim]HSO_4_ as a catalyst. (**a**) sugars in aqueous phase; (**b**) other products in aqueous phase (**c**) organic phase.

**Figure 5:**
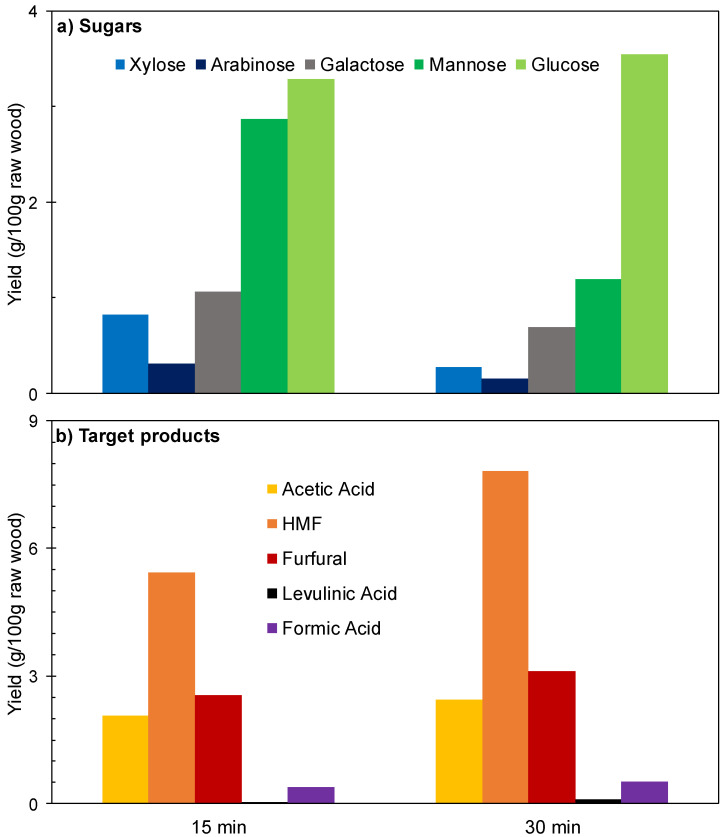
Overall yields (including the contributions of aqueous and organic phases) achieved in experiments performed at 190 °C using [C4mim]HSO_4_ as a catalyst. (**a**) Sugars (**b**) Target products.

Following this idea, new experiments were performed at 190 °C. The data in Figure 4 and Figure 5 show general variation patterns related to the ones observed at 180 °C, with decreased conversions of galactosyl groups into galactose (ascribed to its increased consumption), presence of residual sugars in the reaction media, almost quantitative acetic acid conversion (considering the amounts present in both phases), and increased conversions into furfural (49.7–60.5%), but limited overall conversions of potential substrates into HMF and organic acids. The yields increased significantly at the longest reaction time, but remained below the expected threshold. As before, the experimental data suggested that harsher conditions (for example, operating at higher temperatures, longer reaction times and/or using higher catalyst concentrations) should result in improved results. Alternatively, the utilization of a more active catalyst could serve the same purpose. This latter idea is assessed in the next section.

### 3.3. Experiments Using [C3SO_3_Hmim]HSO_4_ and [C4SO_3_Hmim]HSO_4_

Among the imidazolium-type ILs, the ones having sulfonated alkyl chains possess special properties, including [33,42]: (a) the ability to perform as a solvent and/or as a catalyst, (b) the ability to facilitate the isomerization of glucose into fructose (as a previous step for dehydration into HMF), and (c) the increased acidity caused by the electron-withdrawing nature of the alkyl sulfonic acid side-chain, which is expected to result in increased catalytic activity. Additionally, the catalytic activities of sulfonated ILs bearing the HSO_4_^-^ anion are higher than the ones determined for other ILs made up of the same cation but a different anion [51]. Based on this information and on the results discussed in the previous section, [C3SO_3_Hmim]HSO_4_ and [C4SO_3_Hmim]HSO_4_ were employed as catalysts for manufacturing furans and organic acids from pine wood, following the scheme depicted in Figure 1.

Scarce literature has been reported on the utilization of these AILs for the purposes of this study. Ren et al. [51] employed [C3SO_3_Hmim]HSO_4_ and [C4SO_3_Hmim]HSO_4_ as catalysts to process microcrystalline cellulose in a microwave-heated reactor, reaching an optimal conversion into levulinic acid of 55%. In a related study, Shen et al. [42] reached 39.4% conversion of microcrystalline cellulose in media containing [C4SO_3_Hmim]HSO_4_. Improved results (up to 86.1% cellulose conversion into levulinic acid) were reported by the same research group [51] operating at increased dilutions with a high AIL charge (4 g/g cellulose). In the same study, MIBK was employed to recover levulinic acid from the media after reaction. Teng et al. [49] employed [C4SO_3_Hmim]HSO_4_ in MIBK-containing media for obtaining soluble products (at 71.4% yield) from bagasse, a substrate susceptible to hydrolysis owing to its limited lignin content.

In this study, the experiments with sulfonated ILs were performed at 190 °C (according to the results discussed in the previous section). With respect to the former experiments, the catalyst charge and the relative amount of MIBK remained unchanged, and the reaction time was considered as an operational variable.

**Figure 6:**
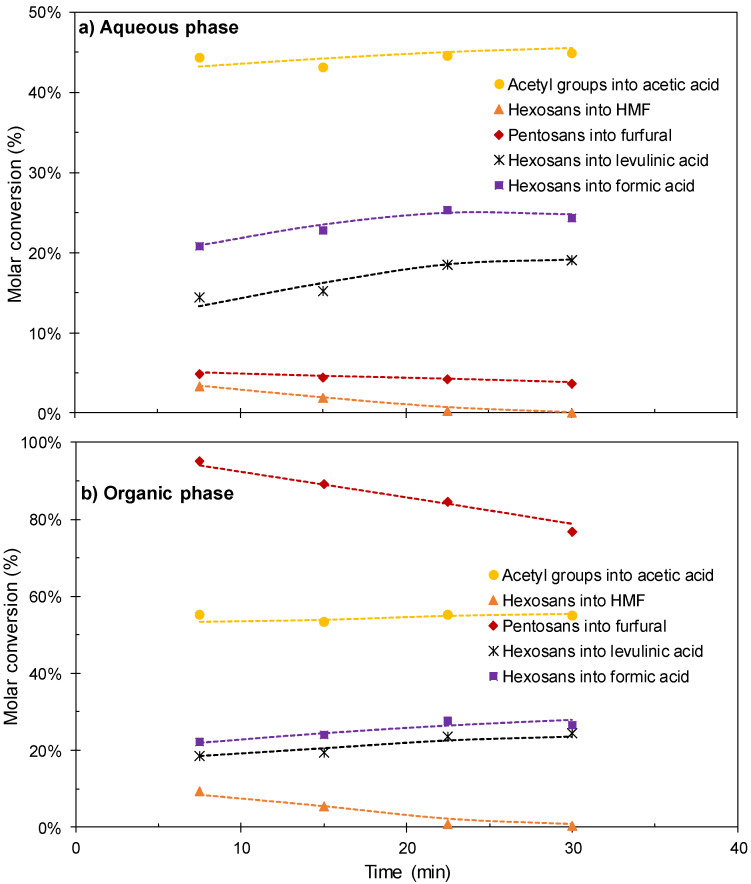
Results achieved in experiments performed at 190 °C using [C3SO_3_Hmim]HSO_4_ as a catalyst. (**a**) compounds in aqueous phase; (**b**) compounds in organic phase.

**Figure 7:**
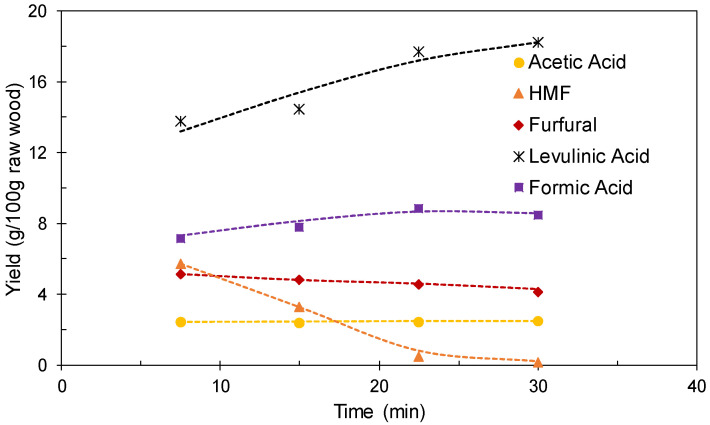
Overall yields (including the contributions of aqueous and organic phases) achieved in experiments performed at 190 °C using [C3SO_3_Hmim]HSO_4_ as a catalyst.

The results obtained in the set of experiments performed at 190 °C using [C3SO_3_Hmim]HSO_4_ as a catalyst are shown in Figure 6 and Figure 7. Figure 6 shows the time course of the conversions of the potential substrates into the target products. The conversion of acetyl groups into acetic acid increased steadily with the reaction time to achieve total conversion under the harshest conditions assayed. The generation of formic acid and levulinic acid from their precursor (hexosans) increased steadily with time up to 22.5 min, but little variation was observed when the reaction time was further increased up to 30 min. Formic acid was produced at a higher conversion rate than levulinic acid, a fact ascribed to generation from sources different from hexoses (i.e., formyl groups in lignin) or to preferential participation of levulinic acid in side reactions. HMF yielded levulinic and formic acids, whereas furfural was obtained at near quantitative yields at the shortest reaction time assayed, and was further consumed in part. The corresponding yields (including the amounts produced in both phases are shown in Figure 7. At reaction times 22.5–30 min, levulinic acid and formic acid were produced at yields of about 18 and 8.6 g/100 g wood, respectively, whereas lower yields of furfural and acetic acid (about 4.3 and 2.4 g/100 g wood, respectively) were obtained owing to the limited wood content of the respective precursors.

A comparative analysis of results indicated that 22.5 min could be considered as the optimal reaction time, owing to the good balance between the opposite trends observed for levulinic acid (concentration increasing with temperature) and furfural (concentration decreasing with temperature). In order to provide additional insight on the fractionation effects reached under these conditions, the spent solid (recovered at a yield of 18.8 g/100 g of oven-dry wood) was subjected to quantitative acid hydrolysis. The experimental value (99.6 ± 0.2% Klason lignin content) confirmed that polysaccharides were totally consumed after 22.5 min.

**Figure 8:**
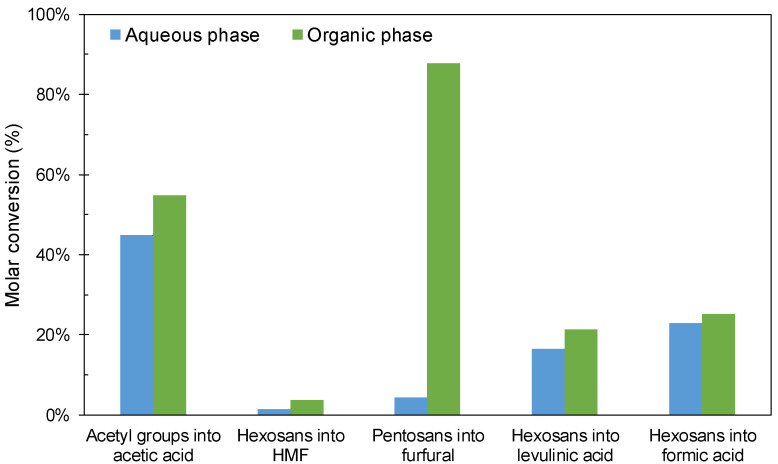
Conversions achieved at 190 °C using [C4SO_3_Hmim]HSO_4_ as a catalyst.

In order to explore the effects of the length of the carbon chain in the AIL cation, [C3SO_3_Hmim]HSO_4_ was replaced by [C4SO_3_Hmim]HSO_4_ in an experiment performed under the conditions identified as optimal for operation with [C3SO_3_Hmim]HSO_4_ (190 °C, 1 g catalyst/10 g oven-dry wood, reaction time = 22.5 min). The results in Figure 8 show that complete conversion of acetyl groups into acetic acid took place (yield 2.43 g/100 g wood), 92% conversion of pentosans into furfural (equivalent to 4.72 g/100 g wood, slightly above the result obtained with [C3SO_3_Hmim]HSO_4_), and 37.8% hexosan conversion into levulinic acid (corresponding to 15.9 g/100 g wood). The results suggest that the increase in length of the AIL cation results in decreased acidity, which is responsible for the slightly slower kinetics of furfural generation/consumption and HMF generation/rehydration into formic and levulinic acids.

## Figures and Tables

**Table 1:** Composition of the *Pinus pinaster* wood lot employed in this study. Data expressed as weight percent, on an oven-dry wood basis.

Component	wt%	Standard Deviation
Glucosyl units	45.1	0.5
Xylosyl units	5.90	0.13
Galactosyl units	1.14	0.03
Arabinosyl units	1.16	0.04
Mannosyl units	12.3	0.2
Acetyl groups	1.74	0.05
Klason lignin	26.8	0.4
Extractives	2.90	0.17
Ash	0.16	0.01
